# Editorial: Rising stars in molecular neuroscience - molecular signalling & pathways: 2022

**DOI:** 10.3389/fnmol.2025.1586932

**Published:** 2025-03-25

**Authors:** Veronika Stoka, Jianhan Chen

**Affiliations:** ^1^Department of Biochemistry and Molecular and Structural Biology, J. Stefan Institute, Ljubljana, Slovenia; ^2^Department of Chemistry, University of Massachusetts Amherst, Amherst, MA, United States

**Keywords:** sensory processing, signaling pathway, ncRNA, synaptic plasticity, neurodevelopment, gene regulation, neurogenesis, intracellular signaling

Neuronal function is regulated by a complex interplay of gene regulatory networks, intracellular signaling pathways, and sensory-driven modulators, which together govern development, synaptic plasticity, and responses to environmental stimuli (Jessell, 2000; Flavell and Greenberg, 2008; Bliss et al., 2014; Yamada et al., 2019). Advances in synaptic plasticity mechanisms (Citri and Malenka, 2008; Sweatt, 2016), non-coding RNA regulation (Salta and De Strooper, 2012; Statello et al., 2021), and receptor-based sensory processing (Dalton and Lomvardas, 2015) have expanded our understanding of how these molecular networks contribute to both neural function and disease progression. Integrating insights into gene regulation, intracellular signaling, and adaptive responses, this Research Topic *Rising stars in molecular neuroscience - molecular signaling and pathways* explores how molecular mechanisms orchestrate neurodevelopment, sensory processing, and injury-induced plasticity.

At the gene regulation level, non-coding RNAs (ncRNAs) have emerged as key regulators of neuronal identity and circuit formation. Wei et al. demonstrated that ncRNAs contribute to the molecular signatures that distinguish different cortical cell types, acting together with other regulatory mechanisms. These findings emphasize the role of gene regulatory mechanisms in defining neuronal circuits and suggest potential implications for neurological disorders involving ncRNA dysregulation.

Beyond transcriptional control, intracellular signaling pathways fine-tune synaptic plasticity and neuronal differentiation. Cherra and Lamb discuss the dynamic interplay between the Ras and Rap small GTPase signaling pathways, historically viewed as antagonistic. Their work highlights the coordinated roles of these pathways, with Ras enhancing synaptic potentiation and Rap contributing to synaptic weakening, which is crucial for maintaining circuit stability. Given the implication of Ras/MAPK signaling dysregulation in neurodevelopmental disorders such as autism spectrum disorder, neurofibromatosis, and intellectual disability, these findings provide valuable insights into potential therapeutic targets for future research.

Regulatory mechanisms extend beyond intracellular signaling to post-translational modifications, which modulate signaling pathways at a finer scale. Hernandez-Lima et al. identified FIC-1 as a key regulator of TGF-β signaling in *C. elegans*, demonstrating that AMPylation of TGF-β disrupts neuronal function and organismal health. Since TGF-β signaling is evolutionarily conserved and plays a role in neuroprotection and synaptic plasticity, these findings suggest that AMPylation may serve as a novel modulator of neural function.

Neurogenesis is another fundamental aspect of brain plasticity that requires precise molecular regulation of neuronal proliferation and integration. Latchney et al. demonstrated that the conditional deletion of PTEN in adult mice initially enhances hippocampal neurogenesis but later leads to spatial dysregulation. While early-stage proliferation is confined to neurogenic regions, over time, newborn neurons expand into non-neurogenic zones such as the hilus and molecular layer, ultimately causing apoptosis and disrupting the hippocampal circuitry. These findings reinforce PTEN's critical role in maintaining the balance between neuronal growth and survival, with direct implications for epilepsy and autism-related macrocephaly.

Beyond intracellular signaling, neurons must integrate external stimuli with internal regulatory networks to ensure appropriate behavioral responses. Sensory processing depends on specialized receptor systems that convert environmental signals into neural activity. Omelchenko et al. investigated temperature sensation in *Drosophila* larvae and identified IR25a and IR93a as key thermosensory receptors. Unexpectedly, they also uncover a direct role for Rhodopsin 1 in cool avoidance. This finding demonstrates that Rh1, a protein traditionally associated with phototransduction, contributes to thermosensation, thus suggesting a complex interplay between different sensory modalities.

A similar compartmentalization of sensory signaling is observed in the retina, where A-Kinase Anchoring Proteins (AKAPs) regulate cAMP/PKA signaling to control visual adaptation. Tomczak et al. showed that AKAPs fine-tune rhodopsin activity in photoreceptors, modulate synaptic plasticity in bipolar cells, influence ganglion cell excitability, and maintain Müller glial ion homeostasis. These findings highlight AKAPs as key molecular organizers, potentially extending their role beyond vision to other sensory modalities.

Following neural injury, the gene regulatory mechanisms shift toward repair and plasticity. Circular RNAs (circRNAs) are emerging as important regulatory molecules in this process. Siddiq et al. investigated the role of circRims2 in axonal outgrowth following spinal cord injury. They found that circRims2 is expressed in axons and its downregulation is correlated with reduced axonal outgrowth *in vitro*. Furthermore, inhibiting ADAR1, an enzyme that suppresses circRNA formation, enhances axonal regeneration after spinal cord injury. These findings suggest that circRims2 and ADAR1 play a role in the molecular mechanisms underlying neuronal regeneration, highlighting the potential of RNA-based therapeutic strategies for neural repair.

Neuropeptides also contribute to sensory-driven adaptation, particularly in pain perception. Takács-Lovász et al. investigated hemokinin-1 (HK-1), a tachykinin-related neuropeptide, and revealed that it modulates pain-related gene expression independently of the NK1 receptor. This suggests an alternative signaling mechanism for HK-1, expanding current models of neuropeptide-based pain regulation and opening new therapeutic possibilities for chronic pain conditions.

In conclusion, the studies featured in this Research Topic illustrate the intricate regulatory networks shaping neuronal function across development, sensory adaptation, and injury response. Emerging leaders' collective findings emphasize how gene regulation, intracellular signaling, and receptor-based modulation sustain neural plasticity and resilience. Specifically, the roles of non-coding RNAs in neuronal identity, Ras/Rap signaling in synaptic plasticity, and circRNA-mediated repair after injury highlight the molecular complexity of neural function in health and disease.

Future research should focus on bridging molecular and systems-level approaches to clarify how these pathways interact within neuronal networks. Advances in single-cell transcriptomics and RNA-based profiling could provide deeper insights into these regulatory mechanisms and their roles in neurological disorders and neurodegeneration. Refining our understanding of these processes will be key to translating molecular discoveries into targeted therapeutic strategies.
